# Enhancing diabetic wound healing: advances in electrospun scaffolds from pathogenesis to therapeutic applications

**DOI:** 10.3389/fbioe.2024.1354286

**Published:** 2024-02-05

**Authors:** Xuewen Jiang, Yu-E Zeng, Chaofei Li, Ke Wang, Deng-Guang Yu

**Affiliations:** ^1^ School of Materials and Chemistry, University of Shanghai for Science and Technology, Shanghai, China; ^2^ Department of Neurology, Ruijin Hospital Lu Wan Branch, Shanghai Jiao Tong University School of Medicine, Shanghai, China; ^3^ Department of General Surgery, Ruijin Hospital Affiliated to Shanghai Jiao Tong University School of Medicine, Shanghai, China

**Keywords:** nanofiber, electrospinning, wound dressing, diabetic foot ulcers, nanostructures

## Abstract

Diabetic wounds are a significant subset of chronic wounds characterized by elevated levels of inflammatory cytokines, matrix metalloproteinases (MMPs), and reactive oxygen species (ROS). They are also associated with impaired angiogenesis, persistent infection, and a high likelihood of hospitalization, leading to a substantial economic burden for patients. In severe cases, amputation or even mortality may occur. Diabetic foot ulcers (DFUs) are a common complication of diabetes, with up to 25% of diabetic patients being at risk of developing foot ulcers over their lifetime, and more than 70% ultimately requiring amputation. Electrospun scaffolds exhibit a structural similarity to the extracellular matrix (ECM), promoting the adhesion, growth, and migration of fibroblasts, thereby facilitating the formation of new skin tissue at the wound site. The composition and size of electrospun scaffolds can be easily adjusted, enabling controlled drug release through fiber structure modifications. The porous nature of these scaffolds facilitates gas exchange and the absorption of wound exudate. Furthermore, the fiber surface can be readily modified to impart specific functionalities, making electrospinning nanofiber scaffolds highly promising for the treatment of diabetic wounds. This article provides a concise overview of the healing process in normal wounds and the pathological mechanisms underlying diabetic wounds, including complications such as diabetic foot ulcers. It also explores the advantages of electrospinning nanofiber scaffolds in diabetic wound treatment. Additionally, it summarizes findings from various studies on the use of different types of nanofiber scaffolds for diabetic wounds and reviews methods of drug loading onto nanofiber scaffolds. These advancements broaden the horizon for effectively treating diabetic wounds.

## 1 Introduction

**FIGURE 1:**
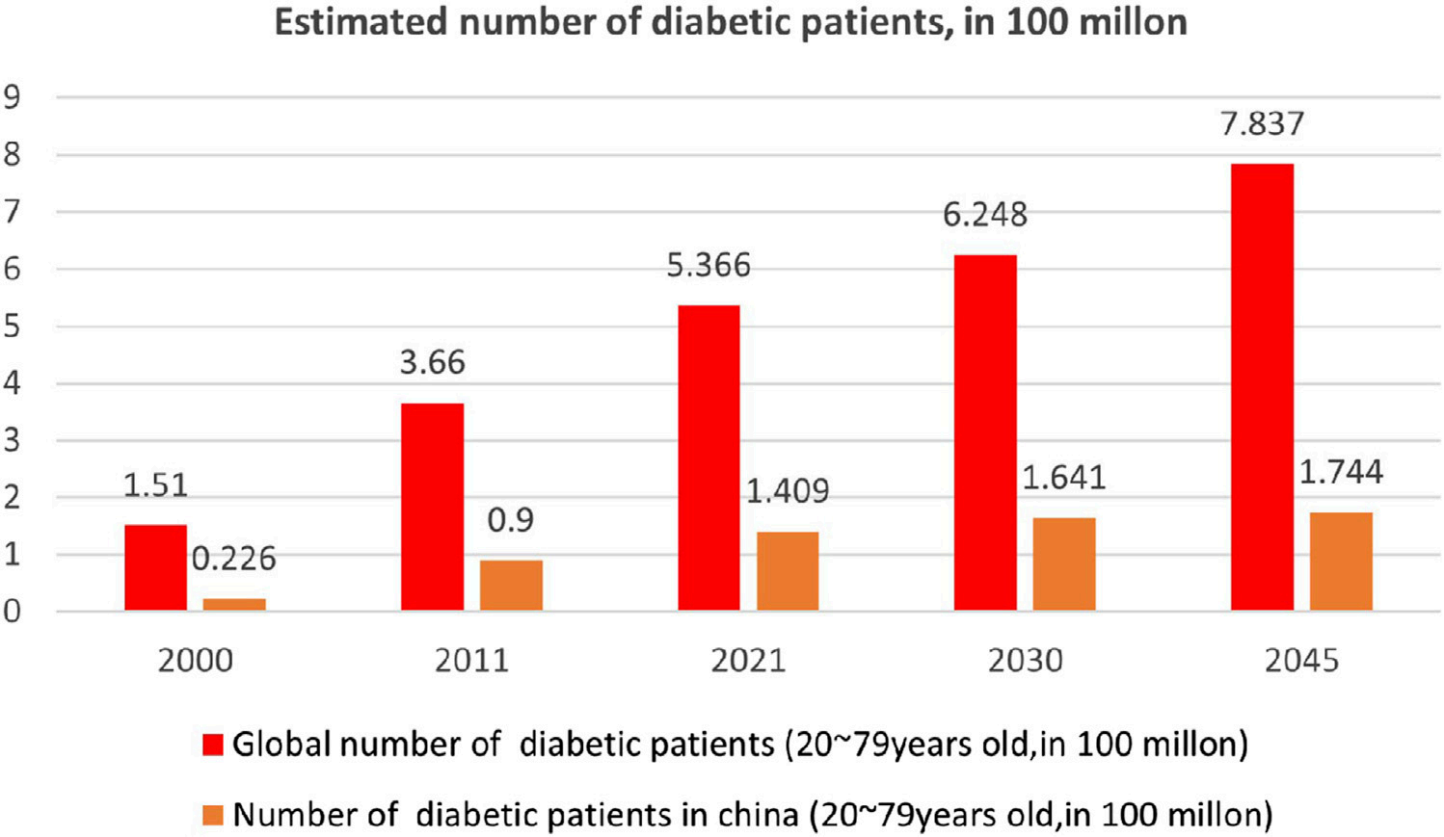
Epidemiological survey of diabetes in China and worldwide (Aschner et al., 2021).

Diabetes mellitus (DM) is a metabolic disease characterized by chronic hyperglycemia resulting from various factors. It occurs due to defects in insulin secretion and/or action. The classic symptoms of DM, referred to as “three polys and one loss,” include polyuria, polydipsia, polyphagia, and weight loss, often accompanied by skin itching. Prolonged metabolic disorders in carbohydrate, fat, and protein metabolism can lead to a range of chronic complications, such as progressive lesions, dysfunction, and failure of vital organs like the eyes, kidneys, nerves, heart, and blood vessels (ElSayed et al., 2023). Severe metabolic disorders can occur during instances of disease severity or stress, contributing to the high global mortality rate associated with diabetes. Consequently, this disease places significant financial strain on healthcare systems worldwide. Type 1 diabetes (T1DM) and type 2 diabetes (T2DM) are the most prevalent forms, both exhibiting evident genetic heterogeneity. Additionally, there are other specific causes of diabetes, such as young-onset adult diabetes and diabetes caused by mitochondrial gene mutations (ElSayed et al., 2023). Despite variations in incidence and pathogenesis among these diabetes subtypes, the underlying pathological basis remains the body’s inability to properly handle and utilize glucose, resulting in elevated blood glucose levels (Tomic et al., 2022). Currently, an estimated 537 million people worldwide are affected by diabetes (Sun et al., 2022). Over the past 2 decades, the global prevalence of diabetes mellitus has significantly increased, primarily driven by diverse social and economic factors. Projections indicate that by 2045, approximately 780 million people will have diabetes, representing an almost 50% increase within the next 20 years (Aschner et al., 2021) (Figure 1).

Diabetic foot ulcer (DFU) is a common and serious complication of diabetes characterized by the development of lower extremity ulcers and gangrene due to macrovascular and microvascular lesions (McDermott et al., 2023). Advancement to the gangrene stage can result in amputation or even death. Peripheral neuropathy and peripheral vascular disease (PAD) are major contributing factors to the development of DFU (Monteiro-Soares et al., 2023). Neuropathy can lead to intrinsic foot muscle contracture, causing claw toe deformity and increased pressure on the metatarsal head, making this area more susceptible to ulcers. Flexion of the proximal interphalangeal joint increases the risk of ulceration on the dorsal side of the joint and the metatarsal side of the toe. Vascular lesions hinder tissue healing, and even minor arterial insufficiency injuries have little chance of healing without vascular reconstruction (Peter-Riesch, 2016). Consequently, DFU is a leading cause of non-traumatic amputations and incurs substantial treatment costs, making it a significant social issue.

Why is DFU therapy expensive? To understand this, we need to examine why wound healing is challenging and what factors prolong the healing process. Peripheral neuropathy, deformities, and macrovascular disease are major contributors to the failure of DFU healing (Veves et al., 2001). Other significant factors affecting DFU include low fibroblast proliferation, receptor downregulation, and inadequate protein matrix in the dermis (Marston et al., 2003). In summary, the mechanisms involved are complex, but they provide ample scope for the treatment of DFU. The treatment approaches for DFU vary based on individual symptoms and disease stage. Regulating blood glucose levels and appropriate insulin therapy have shown high efficacy in wound closure (Veves et al., 2001). Other methods, such as revascularization (Liu et al., 2019), hyperbaric oxygen therapy (Raspovic et al., 2022), and granulocyte colony-stimulating factor (Yellin et al., 2022), have also been used. However, in most cases, wound dressings create an optimal environment for open wounds, leading to better and faster wound closure (Futrega et al., 2014).

However, traditional wound dressings, such as gauze, have limited functionality due to inherent material defects. They can only partially replace the functions of damaged skin and provide a microenvironment for wound healing. Normal wound healing progresses through different stages with distinct pathological characteristics. Therefore, an efficient local administration method is needed to aid in the healing of diabetic wounds. In recent years, new drug delivery systems, including fibrous membranes, foams, and hydrogels, have emerged. However, nanofibers have shown superior results in promoting the formation of new skin tissues, vascular remodeling, and exudate absorption (Gao et al., 2022). The porous structure of nanofibers facilitates the continuous delivery of drugs and allows for easy surface modification to impart specific function (Gao et al., 2021).

Electrospinning technology enables precise adjustment of the composition and size of fibers, making it a versatile tool in various biomedical fields such as medical implants (Singh et al., 2015), wound dressings (Felgueiras and Amorim, 2017), bone tissue engineering scaffolds (Udomluck et al., 2020), antibacterial agents (Mohammadi et al., 2019), drug delivery vehicles (Chakraborty et al., 2009), bionic actuators (Ke et al., 2014), dental materials (Zhao et al., 2022), and enzyme fixation scaffolds (Stojanov and Berlec, 2020). The unique microstructure and appropriate mechanical properties of electrospun scaffolds resemble the nano-network structure of the natural extracellular matrix. This network structure facilitates absorption of wound tissue exudate and promotes gas exchange, enhancing skin tissue regeneration. Additionally, electrospun scaffolds support cell implantation, attachment, nutrient infiltration, and metabolic waste discharge, creating an optimal microenvironment for cell growth, proliferation, adhesion, migration, and differentiation (Kim et al., 2022; Dong et al., 2023; Zhao et al., 2023). Additionally, electrospun scaffolds offer the capability to load bioactive factors or drugs, such as antibiotics, anti-inflammatory agents, hypoglycemic drugs, inorganic nanoparticles, bioactive factors, and specific active cells. Moreover, the fiber structure can be adjusted to achieve precise control over drug release (Qian et al., 2023; Li et al., 2024; Yu and Zhou, 2024). Therefore, electrospun scaffolds hold great promise in the treatment of diabetic foot ulcers (Wang Y et al., 2022).

In this paper, we provide a brief overview of the pathological mechanisms involved in normal and diabetic wound healing, as well as the different structures of nanofiber scaffolds prepared through electrospinning. We then summarize the application of various types of electrospinning scaffolds and electrospinning scaffolds loaded with different drugs in promoting diabetic wound healing.

## 2 Pathology and treatment of diabetic foot ulcers

### 2.1 Pathology of normal wound healing

**FIGURE 2:**
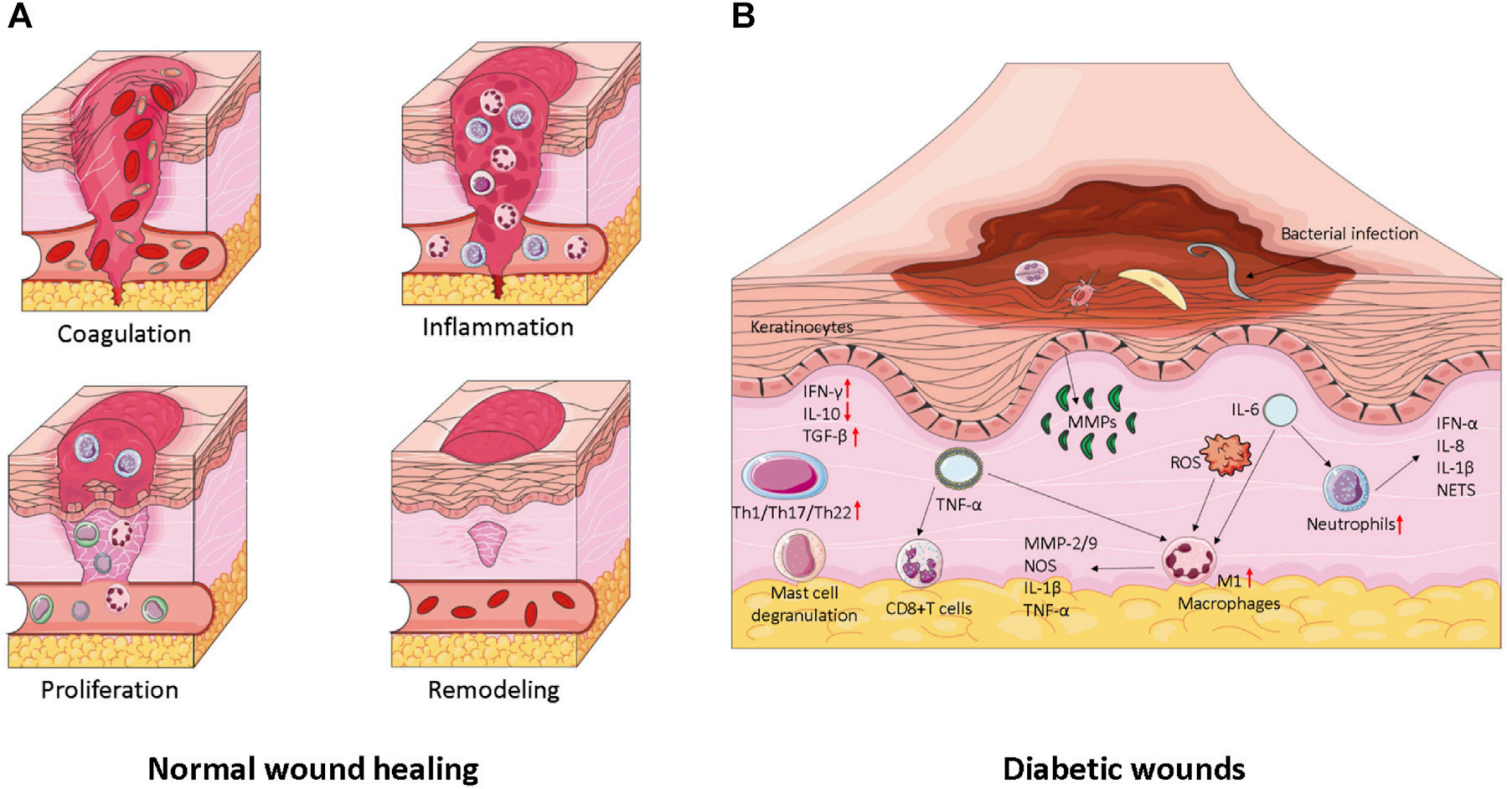
**(A)** Wound healing and its major cellular components. The repair of wounds begins with the coagulation phase, where platelet embolization prevents blood loss and the formation of the initial fibrin matrix. This is followed by the inflammation stage, which serves to remove debris and prevent infection. The influx of neutrophils is promoted by the release of histamine by mast cells. Monocytes then migrate to the site of injury and differentiate into tissue macrophages, which help clear residual cell debris and neutrophils. During the proliferation phase, keratinocytes migrate to close the wound space, angiogenesis occurs to reconstruct blood vessels, and fibroblasts replace the initial fibrin clot with granulation tissue. Macrophages and regulatory T cells play critical roles in this stage of healing. Finally, in the remodeling stage, fibroblasts further remodel the deposited matrix, while vascular degeneration and myofibroblasts contribute to overall wound contraction. **(B)** Factors influencing wound healing in diabetes. Diabetic wound keratinocytes display abnormal activation, leading to impaired hyperproliferation and migration. Additionally, a significant number of chronic inflammatory cells, such as macrophages and fibroblasts, undergo senescence and exhibit a senescence-associated secretory phenotype (SASP). This perpetuates senescence, triggers the release of reactive oxygen species (ROS), and exacerbates inflammation.

Wounds can be classified into two types based on their duration: acute wounds and chronic wounds. Acute wounds, such as surgical wounds, traumatic wounds, and burns, heal rapidly. Chronic wounds, on the other hand, deviate from the normal healing process and persist for more than 3 months. Examples include pressure ulcers, leg ulcers, and diabetic foot ulcers, which require a longer time to heal. General wound healing involves a series of interconnected and complex processes, involving immune cells, formation cells, as well as the cell factors, chemotactic factors, and growth factors they secrete, which coordinate all stages of healing. Traditionally, wound healing is divided into four consecutive stages: hemostasis following skin injury, an inflammatory response lasting several days, tissue regeneration lasting several days to 1 month, and tissue remodeling, as shown in Figure 2A. However, chronic wounds often stall before reaching the late healing stage (Liang et al., 2022).

The first stage of normal wound healing is hemostasis, during which the body rapidly contracts blood vessels through a nerve reflex mechanism to prevent excessive blood loss caused by vascular damage (Sinegre et al., 2019). Blood platelets play a vital role in hemostasis and clotting. They immediately aggregate upon receiving signals from the extracellular matrix (ECM) (Maouia et al., 2020). The ECM then secretes proteins, such as fibronectin and collagen, which attach to platelet receptors and form blood clots (Rojano et al., 2019). Blood clots are specialized structures composed of platelets embedded in various proteins, crucial for preventing bleeding (Yang et al., 2019; Zeng et al., 2020). In the subsequent 1–3 days, the wound enters the inflammatory response phase, during which neutrophils and anti-inflammatory macrophages gradually differentiate at the site of injury (Behm et al., 2012; Decker et al., 2016). These cells are capable of engulfing bacteria that cause infection and removing damaged cellular debris. During the tissue regeneration phase of wound healing, keratinocytes, fibroblasts, macrophages, and endothelial cells differentiate to re-epithelialize cells, reconstruct vascular tissues, and promote fiber proliferation (Lau et al., 2009; Pan et al., 2021). This stage is characterized by the presence of immune cell subsets that facilitate healing, including M2 anti-inflammatory macrophages and regulatory T cells (Tregs). The tissue remodeling stage typically lasts for 1 month but can extend for several months or even years. It primarily involves fibroblasts, which utilize fibrin protein clots to form scar tissue, accelerate neovascularization, and reshape collagen composition (Hinz, 2016). The entire process of normal wound healing begins with platelet aggregation, which forms blood clots at the wound site and secretes bioactive factors to promote immune cell differentiation and maintain blood vessel permeability. Neutrophils and inflammatory macrophages remove bacterial infections, debris, and dead cells through phagocytosis. Subsequently, the pro-inflammatory immune response transitions into a healing-promoting immune response, reducing inflammation and initiating the process of skin tissue remodeling.

### 2.2 Pathology of wound healing in diabetes

Diabetic wounds, including chronic wounds, deep wounds, infected wounds, and ulcer wounds, are characterized by persistent inflammation, recurrent infections, impaired angiogenesis, impaired tissue epithelialization, and excessive levels of matrix metalloproteinases (MMPs) and reactive oxygen species (ROS) (Holl et al., 2021).

Diabetic wounds are one of the three major types of chronic wounds, with the other two being vascular ulcers and pressure ulcers. The hemostatic and inflammatory stages of chronic wound healing are similar to the normal wound healing process. However, unlike other chronic wounds, diabetic neuroischemic lesions and peripheral vascular lesions can significantly impact wound healing. Additionally, there is often a persistent external wound infection, which hinders the transition from the inflammatory response stage to the tissue regeneration stage, thus slowing down the overall healing process (da Silva et al., 2019).

Diabetic complications are commonly accompanied by vascular lesions, leading to nutritional deficiencies in wound healing. The hypoxic environment of the wound further complicates angiogenesis, and the wound remains in a chronic state of inflammation (Peng et al., 2021). During general wound healing, fibroblasts, keratinocytes, and immune cells secrete MMPs under the influence of local mediators. These mediators, such as transforming growth factor-beta (TGF-β), vascular endothelial growth factor (VEGF), epidermal growth factor (EGF), and interleukins, are essential for tissue epithelialization and regeneration. However, in the high glucose environment of diabetes, the activation of the extracellular regulated protein kinases/activating protein-1 (ERK/AP1) signaling pathway can lead to excessive production of MMPs, resulting in rapid degradation of the extracellular matrix (ECM) (Roep et al., 2019). This affects the adhesion of fibroblasts to the ECM, hindering the regeneration of skin tissue. Activated neutrophils can produce large quantities of matrix metalloproteinase 9 (MMP-9), further impeding the repair of diabetic ulcers (Nguyen et al., 2018). Under normal circumstances, macrophages transition from the M1 type that promotes inflammation to the M2 type that promotes wound repair under the influence of signaling factors, thus reducing inflammation. However, in diabetic wounds, macrophages fail to differentiate into the M2 type and instead produce pigment epithelium-derived factor (PEDF), which inhibits neovascularization. Additionally, neutrophils and M1 macrophages persist in the wound, sustaining inflammation and hindering the regeneration of skin tissue. Moreover, the high blood glucose environment in diabetic wounds promotes bacterial proliferation, leading to recurrent infections (Schreml et al., 2010).

### 2.3 Pathogenesis of diabetic foot ulcers

Over time, diabetes can lead to microvascular and macrovascular complications, causing significant pain and suffering for patients. These complications are important factors in the development and progression of diabetic foot ulcers (DFUs). Additionally, immune damage, reactive oxygen species (ROS) production, and bacterial infections also contribute to the development of DFUs (Madhukiran et al., 2021). Diabetic neuropathy (DN) is another complication that can lead to the development of DFUs in diabetic patients (Feldman et al., 2019). The hyperglycemic environment in diabetes often disrupts the arrangement of Schwann cells and axons, leading to impaired peripheral nerve function in DN patients. This disruption also affects the microcirculation of the limbs, resulting in additional nerve damage and loss of foot pain perception (Gumy et al., 2008). The absence of pain sensation makes patients unaware of wounds or ulcers, leading to their enlargement and subsequent infection (Feldman et al., 2019). DN can damage axons, disrupt foot circulation, cause peripheral arterial disease (PAD), and further worsen the condition (Megallaa et al., 2019, p. 2).

During the inflammatory response stage of DFUs, the phagocytic function of macrophages is impaired, resulting in an abundance of M1 macrophages and a prolonged inflammatory state in diabetic wounds. This leads to continuous release of matrix metalloproteinases (MMPs) and rapid degradation of collagen and the extracellular matrix (ECM) (Zhao et al., 2017). Furthermore, the polarization of M1 macrophages slows down the rate of re-epithelialization (Huang et al., 2019). DFUs produce excessive reactive oxygen species (ROS), leading to increased oxidative stress (Singh et al., 2008). These excess ROS react with nitric oxide (NO) to generate peroxynitrite anion (NO_2_^−^), which interferes with wound healing (Sadati et al., 2018). Moreover, in a hyperglycemic environment, the increased ROS levels promote the formation of advanced glycation end products (AGEs) (Kim and Kwon, 2014). Additionally, hyperglycemia increases glycosylation and enhances intercellular interactions in keratinocytes, resulting in delayed wound closure (Huang et al., 2017) (shown in Figure 2B).

### 2.4 Therapeutics for diabetic foot ulcers

Early control in the development of DFU is crucial for successful treatment, similar to many critical illnesses. Traditional wound healing strategies can be categorized into four aspects: unloading, blood glucose control, debridement, and infection management.

DFUs typically occur in high-pressure areas of the foot, underscoring the importance of pressure redistribution (unloading) during DFU therapy. Various methods can be employed to reduce stress, including the use of canes, wheelchairs, surgical interventions, surgical shoes, bandages, and more. Total Contact Castings (TTC) made from specialized materials are widely regarded as the most effective method for unloading during the DFU healing process. Compared to dressings, TTCs have been shown to significantly shorten the healing time of DFUs (Sahu et al., 2018). However, TTCs also have notable disadvantages. They require the expertise of well-trained staff and patient compliance can be poor. In many cases, they cannot be successfully removed, and improper use can even worsen the condition.

Long-term hyperglycemia in diabetic patients can have a detrimental impact on DFU healing, making glycemic control necessary. Glycemic control is typically achieved through medication and a controlled diet to maintain normal blood glucose levels.

Debridement is an essential component of standard DFU care, as it promotes wound healing by removing necrotic tissue and reducing bacterial biofilm and excess matrix proteins. Various types of debridement exist, with surgical debridement being the most commonly used. However, one must be cautious with debridement as it requires time and multiple procedures to yield positive therapeutic effects. A slight oversight can lead to the rapid and severe progression of diabetic foot conditions.

In patients with DFUs who progress to the point of requiring amputation, the ulcerated area often presents with severe infection (Huang et al., 2022b). This is primarily due to the prolonged exposure of the wound in diabetic patients. Infections are typically managed with orally administered antibiotics, which pose significant limitations to treatment (Zhang et al., 2023). High antibiotic concentrations can have serious side effects on the human body. Therefore, it is imperative to explore new strategies for DFU treatment and management (Hu et al., 2021).

## 3 Nanofiber scaffolds with various structures prepared by electrospinning

### 3.1 Introduction to electrospinning technology

The treatment of diabetic wounds and DFUs involves debridement, improved glucose regulation, infection control, and the use of moist dressings to facilitate wound healing and restore blood supply (Tottoli et al., 2020). However, when these traditional treatments prove inadequate, new therapeutic approaches are sought. In recent years, our understanding of diabetic wounds has expanded, paving the way for the development of novel multi-functional wound scaffolds (Al-Dhahebi et al., 2022; Yu and Zhao, 2022; Si et al., 2023).

**FIGURE 3:**
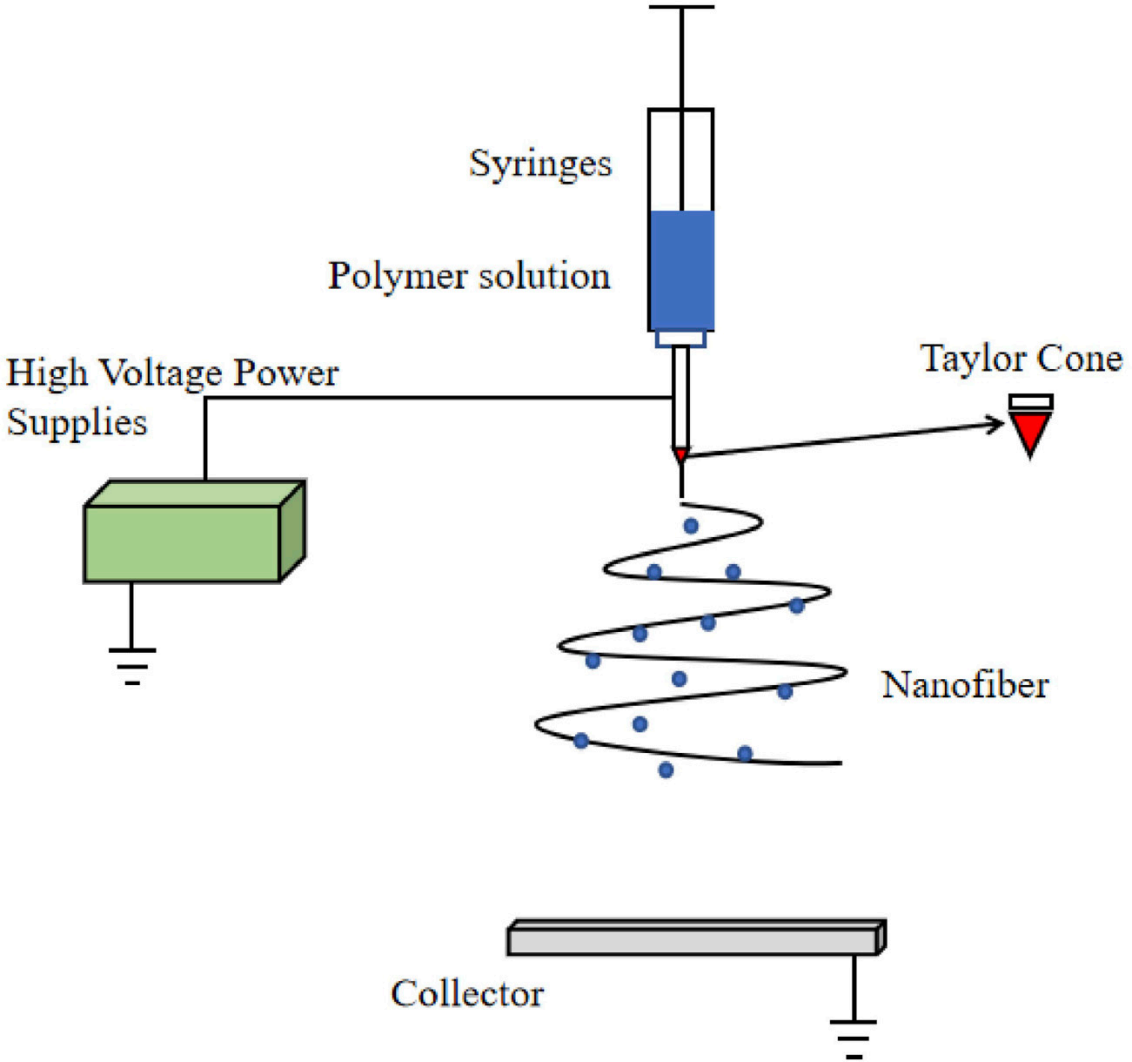
Schematic diagram of electrospinning equipment.

Among these approaches, electrospinning technology is noteworthy. It is a well-established process for fabricating nanofibers (Wang H et al., 2023), with a history spanning several centuries. By carefully adjusting the process parameters of the electrospinning equipment (such as receiving distance, voltage, and flow rate), environmental variables (including humidity and temperature), and polymer solution variables (such as viscosity, solution electrical conductivity, capillary force, molecular weight, and polymer type), electrospun nanofibers with desirable physical properties and excellent structures can be obtained. Electrospinning equipment typically consists of four components: a high voltage power supply, an injector, a spinning nozzle, and a receiving screen (as illustrated in Figure 3). The underlying principle involves applying an electrostatic field of tens of thousands of volts between the injection device and the receiving device, resulting in the formation of a jet from the tapered end of the spinning solution. This jet is then stretched within the electric field and ultimately forms a nonwoven nanofiber structure on the receiving device (Kang et al., 2020).

### 3.2 Advantages of electrospun nanofiber scaffolds in diabetic wound healing

One of the main advantages of electrospinning is the ability to create highly porous fiber structures that mimic the extracellular matrix (ECM) and that aid in wound healing (Sell et al., 2010). this makes it an excellent choice for the fabrication of DFU therapeutic scaffolds (as shown in Table 1). By optimizing the processing and environmental parameters of electrospinning, the porosity and pore size of the scaffolds can be tuned to promote diffusion. This porous structure facilitates gas exchange at the wound site and can also serve as a platform for drug loading, allowing for controlled drug release through structural adjustments (Samadian et al., 2020).

**TABLE 1:** Comparison of electrospun nanofiber scaffolds and extracellular matrix (ECM) (Sell et al., 2010).

	ECM	Electrospun scaffolds
Components	ECM composition consists of a dynamic three-dimensional arrangement of polysaccharides and natural polymers (collagen, elastin, fibrinogen, etc.)	Electrospun scaffolds are mostly prepared from synthetic and natural polymers
Structures	The ECM structure consists mainly of fibres with diameters between 50 and 500 nm	Most of the nanofibres prepared by the electrostatic spinning technique have diameters ranging from 10 μm to 500 nm
Role in wound healing	i. During development, cell-ECM interaction is responsible for pattern formation, morphogenesis, and phenotype acquisition and maintenance	i. Have appropriate porosities to allow for cellular migration and penetration
	ii. During clot formation, wound healing, inflammation, formation of granulation tissue, and remodeling are all mediated by cell-ECM interaction	ii. Have sufficient surface area and the proper surface chemistry to promote cell adhesion, growth, migration, and differentiation
	iii. Cell adhesion, migration, growth, differentiation and apoptosis are all based on a structural framework of tissue composed of ECM.	iii. A rate of degradation that closely matches the rate of native tissue regeneration to promote proper tissue ingrowth

The constructed scaffold exhibits a high specific surface area and volume ratio, resulting in increased drug loading capacity and consistent drug release. Moreover, its porous nature promotes wound desiccation, creating an ideal healing environment (Junker et al., 2013). Electrospinning can produce nano-scale scaffold fibers, increasing the surface area available for cell adhesion and facilitating wound healing (Glover et al., 2021).

To effectively treat DFU, it is important to focus on vascularization, collagen accumulation, and normal physiological function. This will help control the deterioration process and promote wound healing (Lv et al., 2017). Although DFU patients often struggle to heal on their own, biomolecules and active drugs, such as antibiotics, can provide much-needed assistance. Electrospun nanofiber scaffolds are an excellent platform for carrying active drugs due to their controlled-release capabilities. Additionally, nanofiber scaffolds can positively influence exudate uptake and exchange of oxygen, water, and nutrients, which are vital activities in wound healing (Kim et al., 2012).

**FIGURE 4:**
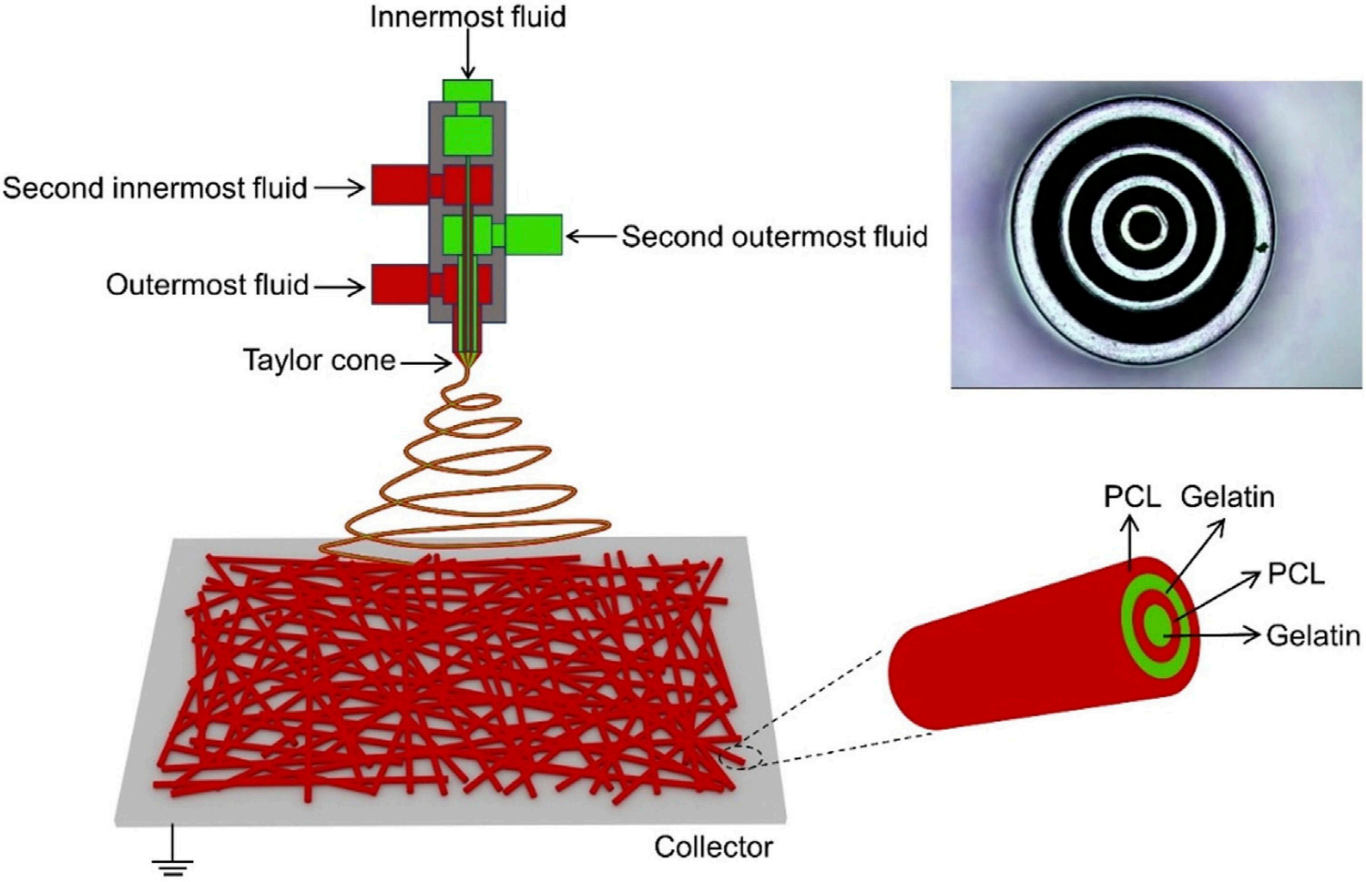
Schematic diagram illustrating quadriaxial electrospinning, with the inset in the upper right corner displaying a photograph of the concentric spinneret (Zhang et al., 2021).

Different types of electrospun scaffold structures require specific preparation methods, including single-fluid and two-fluid electrospinning, as well as the emerging multifluid electrospinning (as shown in Figure 4). Scaffolds prepared using these methods possess unique advantages for wound healing applications. For instance, Ren et al. (Han et al., 2019) investigated the role of oriented scaffolds prepared by single-fluid electrospinning in wound healing and observed a significant enhancement in cell adhesion. Ahmed et al. (Ahmed et al., 2018) developed a zinc oxide (ZnO)-loaded chitosan/polyvinyl alcohol core-shell nanofiber scaffold using electrospinning technology. The antibacterial efficacy and antioxidant potential of both ZnO-loaded and ZnO-free scaffolds were evaluated, demonstrating excellent antibacterial and antioxidant properties. These scaffolds, which exhibited antibacterial properties against both Gram-positive and Gram-negative bacteria, were further tested for wound healing in rats, both with and without cells. The results showed successful wound healing and complete epithelial regeneration (Ranjbar-Mohammadi et al., 2016). Coaxial electrospinning technology was employed to fabricate a core-shell scaffold composed of chitosan and polylactic acid, which improved its mechanical properties and enhanced cell adhesion and proliferation. This scaffold has found applications in tissue engineering (Surucu and Sasmazel, 2016). Furthermore, electrospun scaffolds can be combined with 3D printing techniques to replicate the ECM matrix structure, thereby promoting wound healing in diabetes (Mellor et al., 2017; Zhang et al., 2021).

## 4 Application of electrospun scaffolds for diabetic foot ulcers

The nanofiber scaffolds fabricated through electrospinning technology have demonstrated exceptional properties for wound healing. Their tissue structure closely resembles the extracellular matrix (ECM) found in human skin, thereby promoting the regeneration of new skin. Additionally, these scaffolds serve as versatile platforms for drug loading and delivery, enabling the targeted delivery of wound-healing agents to expedite the healing process. Consequently, electrospun scaffolds are considered ideal materials for treating chronic wounds (Jeckson et al., 2021). Given that diabetic wounds represent a significant subset of chronic wounds, the application of electrospun scaffold materials holds immense potential in the field of diabetic wound healing (Zhou et al., 2023b) (as shown in Table 2).

**TABLE 2:** Advantages and disadvantages of different electrospinning structures. (Wang Y et al., 2023; Chen et al., 2024; Duan et al., 2024).

Technique	Polymer	Active ingredients	Highlights	Ref.
Blend	CS/PVA	Ursolic acid	CS-PVA-UA nanofiber scaffolds are morphologically similar to the natural skin extracellular matrix and have excellent surface hydrophilicity, wettability, and hemostatic properties	Lv et al. (2022)
	Polyvinyl alcohol/PVA	Chitosan	The addition of PCL improved the mechanical and biological properties of the scaffolds	Gholipour-Kanani et al. (2016)
	GZS	Cu	Graphene oxide enhanced mechanical stability and swellability of the scaffold, and copper ions were incorporated as a bactericide and angiogenesis promoter	El-Lakany et al. (2020)
	PVA	EPP1	PVA/EPP1 fiber scaffolds exhibited excellent water absorption and anti-inflammatory activity	Guo et al. (2022)
	TE/collagen	—	Makes the new skin on the wound closer to the natural uninjured skin	Kellar et al. (2020)
	PVA/calcium alginate	Idebenone	the scaffold promoted new skin formation while reducing oxidative stress and inflammation	Jintao (2022)
Coaxial	PVA/PCL	DOX	Achieved continuous release of DOX in response to pH by utilizing the sensitivity of PVA to pH	Yan et al. (2020)
	PLGA	Insulin	The scaffold releases insulin for up to 4 weeks	Lee et al. (2020a)
	PLA/PVA	CTGF	PLA/PVA scaffolds have high biocompatibility and biosafety.the scaffolds can help with vascular remodeling	Augustine et al. (2019)
	PLGA	PDGF, vancomycin	The scaffold could stably release PDGF and antibiotics for up to 3 weeks, enhanced neovascularization around the wound area	Lee et al. (2020b)
	TE/collagen	—	Makes the new skin on the wound closer to the natural uninjured skin	Kellar et al. (2020)
Janus	PVP/EC	TAM	During the initial stage of drug release, TAM-PVP demonstrated a more rapid burst release and a slower sustained release in the subsequent phase, whereas TAM-EC exhibited the opposite pattern	Zheng et al. (2021)
Three-layered	PCL/gelatin/PLGA	BSA/RHB	A novel and robust trifluid electrospun fibrous scaffold was developed with dual drug delivery capabilities	Nagiah et al. (2020)
	Eudragit S100	Aspirin	The nanofibers exhibited a stable and sustained release of the drug, thereby avoiding the risk of excessive administration	Ding et al. (2020)
	CA	ACY	The drug release profile exhibited a consistent and controlled release of ACY throughout the testing period	Wang et al. (2020)
	PCL/collagen	lignocaine extract	These findings indicate that the herbal extracts did not affect the physical properties of the scaffolds, but significantly augmented their bioactivity, making them valuable for the treatment of diabetic ulcers	Derakhshan et al. (2022)

### 4.1 Uniaxial nanofiber scaffold for diabetic wounds

Uniaxial nanofiber scaffolds are typically prepared through blend electrospinning, where the drug and polymer solutions are mixed and electrospun through a single nozzle. Traditional single-axis electrospinning has garnered significant attention from researchers due to its simplicity, cost-effectiveness, high yield, and ease of operation. However, blend electrospinning also has its drawbacks, as it requires the polymer to be spinnable (Wu et al., 2020). By combining two different polymers through blend electrospinning, uniaxial nanofiber scaffolds can be prepared, inheriting the advantageous properties of both polymers, such as good mechanical properties and biocompatibility. However, it is possible for these scaffolds to exhibit deficiencies in certain properties, which can be improved by incorporating additional polymers or utilizing physical techniques such as cross-linking or lyophilization.

For instance, Lv et al. (2022) successfully fabricated a series of blend electrospun nanofibers by combining chitosan (CS) and polyvinyl alcohol (PVA) with the addition of ursolic acid (UA). The resulting CS-PVA-UA nanofiber scaffolds exhibited a morphology resembling collagen fibers in the natural skin extracellular matrix, along with excellent surface hydrophilicity, wettability, and hemostatic properties. The addition of UA to CS-PVA nanofibers also demonstrated good biocompatibility and drug release properties. *In vivo* experiments conducted on diabetic mice showed that the nanofiber scaffold effectively inhibited inflammation and oxidative stress, promoted angiogenesis, collagen deposition, epithelial formation, and hair follicle regeneration, ultimately achieving wound healing. Gholipour-Kanani et al. (2016) prepared chitosan-polyvinyl alcohol nanofiber scaffolds, and subsequently incorporated polycaprolactone (PCL) to enhance mechanical and biological properties. These scaffolds were tested in both a diabetic rat back skin wound model and a diabetic foot ulcer wound model. The results showed significant reduction in wound area and increased new granulation tissue formation in rats treated with the nanofiber scaffolds. Complete wound closure was achieved within 20 days of resection. The addition of PCL improved the mechanical and biological properties of the scaffolds.

El-Lakany et al. (2020) introduced a copper-grafted graphene oxide cross-linked zein electrospun scaffold (Cu-GZS) for promoting healing of skin excision wounds in diabetic male rats. Graphene oxide enhanced mechanical stability and swellability of the scaffold, and copper ions were incorporated as a bactericide and angiogenesis promoter. The optimized Cu-GZS exhibited a constant copper release rate and accelerated wound closure, as evidenced by reduced leukocyte infiltration, intact epithelium with normal keratinization, and faster wound size reduction. Guo et al. (2022) developed a nanofibrous scaffold composed of marshmallow polysaccharide (EPP1) and polyvinyl alcohol (PVA), which exhibited excellent water absorption and anti-inflammatory activity. *In vitro* and *in vivo* experiments demonstrated that PVA/EPP1 fiber scaffolds accelerated wound repair in diabetic mice. Kellar et al. (2020) investigated the synthesis of electrospun biomimetic scaffolds containing Tropoelastin (TE) and collagen. *In vivo* experiments using a diabetic mouse model revealed that the scaffold group exhibited increased neoplastic skin tissue, faster wound healing, reduced inflammatory response, and closer resemblance to unwounded skin compared to the control group. Jintao (2022) prepared PVA/calcium alginate scaffolds through blend electrospinning and loaded idebenone at different concentrations for diabetic wound healing. The scaffold loaded with 1% idebenone demonstrated the best performance. *In vivo* evaluation using a rat diabetic wound model showed that the scaffold group achieved the fastest wound closure rate. Histological immunostaining experiments revealed that the scaffold promoted new skin formation while reducing oxidative stress and inflammation.

### 4.2 Multifluid electrospun nanofiber scaffold for diabetic wounds

Due to the limitations of uniaxial nanofiber scaffolds, it is difficult to prepare more complex nanostructures. As a result, a multi-fluid electrospinning technique has been developed, in which two or more solutions are pushed into a spinneret using various syringes for electrospinning. The main types of multi-fluid electrostatic spinning processes are coaxial electrostatic spinning, side-by-side electrostatic spinning, and triaxial (three-layer coaxial, three-layer side-by-side) electrostatic spinning.

Among the various electrospun nanofibers, the core-shell nanofiber is the most widely used (Rathore and Schiffman, 2021). Compared with other nanofibers (as shown in Table 3), the core-shell nanofiber scaffold can delay drug release and reduce its severity (Wang Y et al., 2023; Chen et al., 2024; Duan et al., 2024). To prepare nanofiber scaffolds with a core-sheath structure, two spinnerets of different apertures are used to nest into a concentric circle-like spinneret. The drug is loaded into the core layer, and the shell layer can slow down the release of the drug. Additionally, two different drugs can be loaded in this core-shell structured nanofiber, and the drugs can be released sequentially. The advantages of the core-shell type nanofiber structure are that it can achieve different sustained-release or quick-release effects based on the adopted macromolecules, and it can conveniently meet the transportation requirements of different drug (Han and Steckl, 2019). Therefore, it is considered to be one of the important breakthroughs in the drug delivery system (Pant et al., 2019; Yu and Huang, 2023). Yan et al. (2020) prepared a PVA/PCL core-shell nanofiber scaffold loaded with doxorubicin (DOX) using electrospinning technology and achieved continuous release of DOX in response to pH by utilizing the sensitivity of PVA to pH.

**TABLE 3:** Summary of different electrospun nanofibre scaffolds in the treatment of diabetic foot.

Double-fluid electrospinning		Multifluid electrospinning
Coaxial electrospinning	Side-by-side electrospinning	Three-fluid electrospinning
i. The drug is loaded into the core layer, and the drug is effectively protected from the external environment through the shell layer	i. Both sides of the polymer material and the drug are in direct contact with the outside world	Triaxial electrospinning is an improved process based on coaxial and juxtaposition electrospinning, which share their common advantages and are capable of constructing more complex drug controlled release systems
ii. By adjusting the composition and thickness of the fibres of the core-shell layer, the function of controlling the slow release of the drug is achieved		
iii. Encapsulate different drug molecules in the core-sheath layer separately to prepare a multistage multi-effect drug release system	ii. It is able to constitute a dual-drug biphasic release drug release system, which helps the drug to be released rapidly	
iv. The non-spinable polymer can be used as the core layer and the spinnable polymer as the shell layer, so that the shell layer can drive the core layer to electrospin, and the non-spinable polymer nanofibres can be prepared		
i. At best, nanofibres capable of controlled release of two drugs have been prepared, which does not fully satisfy more complex and difficult therapeutic requirements	i. It is difficult to achieve the slow drug release effect of coaxial electrospinning	The preparation process is very difficult and requires a level by level modulation of parameters, with all the polymers and drugs affecting the preparation
ii. The preparation process is more complex and the parameters are more difficult to regulate compared to single-fluid electrospinning	ii. Similarly, juxtaposition electrospinning does not meet more complex and difficult therapeutic needs. It is also more complicated in the preparation process	

Lee et al. (2020a) developed insulin-loaded poly-D-L-lactic-co-glycolic acid (PLGA) core-shell nanofiber scaffolds that can stably release insulin to reduce blood sugar and aid in diabetic wound healing. They placed PLGA and insulin solutions into two syringes and electrospun them through two different injection pumps. The prepared scaffold releases insulin for up to 4 weeks. Additionally, the hydrophilicity of the core-shell scaffold is better than that of the mixed nanofiber scaffold. Gene expression detection showed that compared with the control group, type I collagen decreased and transforming growth factor-β increased in the scaffold group. This demonstrates the benefit of the core-shell scaffold, which allows for the delayed release of medication and aids in wound healing. Augustine et al. (2019) developed an electrospun dual porous polylactic acid/polyvinyl alcohol (PLA/PVA) nanofiber scaffold loaded with connective tissue growth factor (CTGF). CTGF was loaded into the polyvinyl alcohol sheath. The scanning electron microscope (SEM) results showed high porosity in the scaffold with secondary pores on the surface of many single fibers. Cell experiments demonstrated that PLA/PVA scaffolds have high biocompatibility and biosafety. The drug release curve also showed stable release of CTGF. Chicken chorioallantoic membrane (CAM) experiments further demonstrated that the scaffolds can help with vascular remodeling. Lee et al. (2020b) developed a PLGA nanofiber core-shell scaffold loaded with antibiotics and platelet-derived growth factor (PDGF). In this scaffold, PDGF acts as the outermost shell, while the core layer consists of a mixture of PLGA and vancomycin. Drug release experiments showed that the scaffold could stably release PDGF and antibiotics for up to 3 weeks. *In vivo* experiments showed that the scaffold enhanced neovascularization around the wound area.

Gupta and Wilkes published the first study on the fabrication of Janus nanofibers by side-by-side electrospinning in 2003 (Gupta and Wilkes, 2003). Side-by-side electrostatic spinning involves combining two spinnerets side-by-side to prepare Janus fibers made of two materials that can both come into direct contact with the environment (Li et al., 2019; Lv et al., 2023a; Lv et al., 2023b). Zheng et al. (2021) used a side-by-side electrospinning procedure to create two Janus nanofiber scaffolds. The first scaffold combined tamoxifen citrate (TAM) as the medicine and ethylcellulose (EC) as the polymer carrier matrix. The second scaffold, TAM-PVP, used TAM as the medication and polyvinylpyrrolidone (PVP) K60 as the polymer carrier matrix. Due to differences in PVP and EC solubilities, drug release tests demonstrated that biphasic drug release was feasible for the two groups of fibers. However, the drug release characteristics of the two fiber groups differed dramatically. During the initial stage of drug release, TAM-PVP demonstrated a more rapid burst release and a slower sustained release in the subsequent phase, whereas TAM-EC exhibited the opposite pattern. This demonstrates the effectiveness of the Janusnanofiber scaffold in achieving different drug release profiles (Hussein et al., 2022).

**FIGURE 5:**
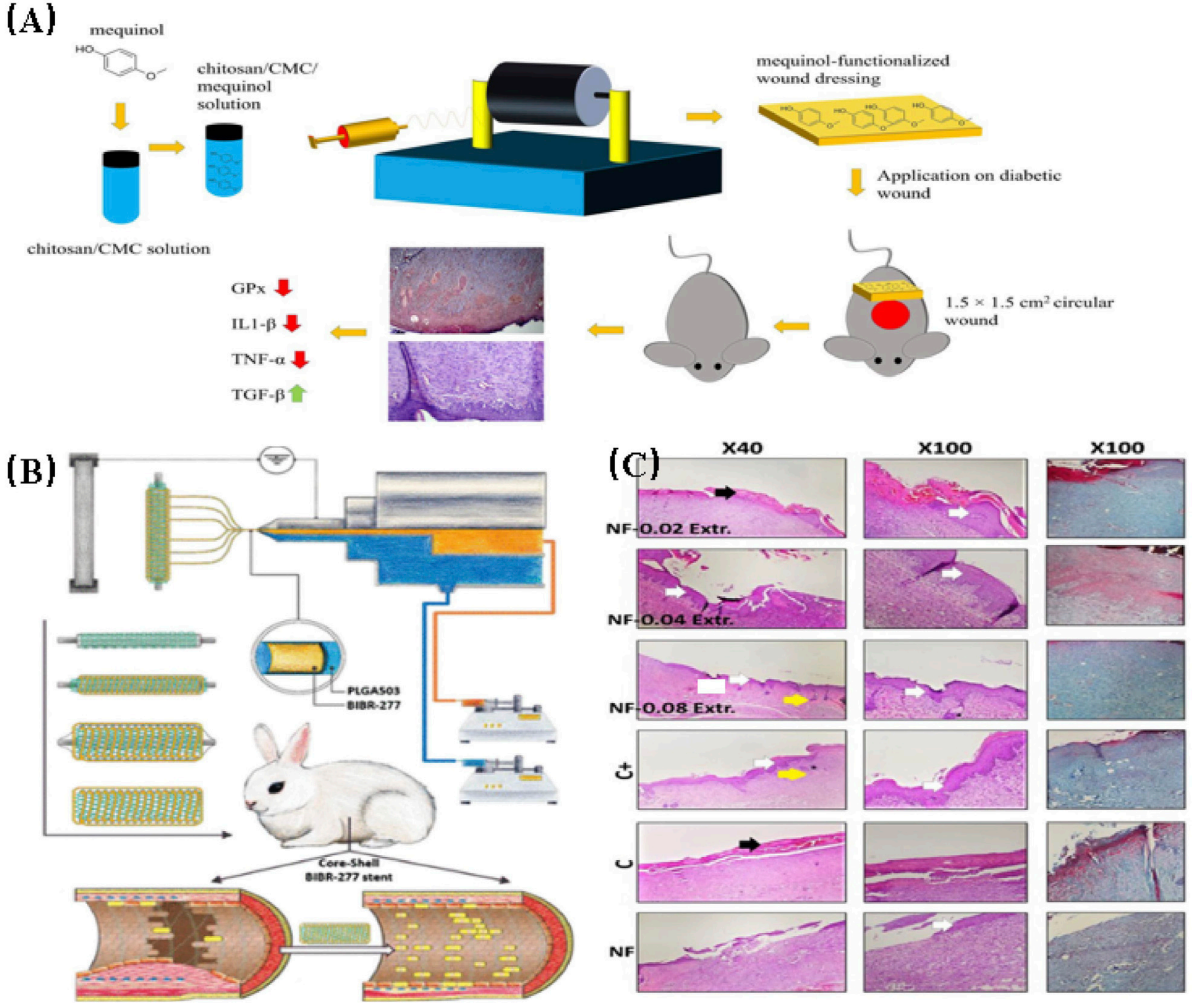
**(A)** A schematic diagram illustrating the development of a drug delivery wound dressing by incorporating methaqualone into the matrix of an electrospun chitosan/carboxymethyl cellulose (CMC)-based scaffold (Abdelbasset et al., 2022). **(B)** Schematic diagram depicting the construction of biodegradable core-shell nanofibers for the continuous and localized delivery of BIBR-277 to the wall of a balloon-injured artery in the abdominal aorta of diabetic rabbits (Lee et al., 2022). **(C)** Microscopic sections of skin samples from wounds following an 18-day treatment period, stained with Hematoxylin and eosin (H&E) and Masson’s trichrome (MT). Black arrows indicate crusty scabs, white arrows indicate the epithelial layer, yellow arrow indicates hair follicle, and asterisks indicate Sebaceous gland (Derakhshan et al., 2022).

Multifluid electrospinning, including the trifluid electrospinning method, is an advanced technique that enhances the electrostatic spinning process by incorporating multiple solutions. One notable application of this technique is the fabrication of core-shell nanofibers, which holds great potential for the treatment of diabetic wounds (Zhou et al., 2023c). In trifluid electrospinning, three fluids are simultaneously electrospun, resulting in nanofibers with a gradient structure consisting of three layers. The medication is typically loaded in the innermost layer, ensuring controlled and uniform release of the drug (Huang et al., 2022a). As a result, the release rate remains constant, eliminating the possibility of abrupt or excessive drug release. In a study conducted by Nagiah et al. (2020), a novel and robust trifluid electrospun fibrous scaffold was developed with dual drug delivery capabilities. The scaffold consisted of a core layer made of PCL, a middle layer composed of gelatin loaded with bovine serum albumin (BSA) as the therapeutic drug, and a shell layer made of PLGA loaded with rhodamine B (RHB). The resulting nanofibers exhibited superior mechanical properties and drug release capabilities compared to both uniaxial and coaxial fibers. Ding et al. (2020) employed a modified triaxial electrospinning technique to fabricate core-shell nanofibers using Eudragit S100 (ES100) as the base material, which was loaded with aspirin. The *in vitro* drug release experiments demonstrated that these nanofibers exhibited a stable and sustained release of the drug, thereby avoiding the risk of excessive administration. In a similar vein, Wang et al. (2020) developed a novel approach for the preparation of three-layer nanoreservoirs and the construction of three-level core-sheath nanofibers. The drug components comprised cellulose acetate and acyclovir (ACY). The drug release profile exhibited a consistent and controlled release of ACY throughout the testing period. Derakhshan et al. (2022) conducted a study on a three-layer electrospun scaffold composed of PCL, PCL/collagen, and collagen. The collagen layer was supplemented with lignocaine extract, known to enhance the healing of diabetic wounds by promoting vascularization. Scanning electron microscopy analysis confirmed the favorable morphology of the nanofibers. In order to evaluate the wound healing efficacy of the fabricated scaffolds, streptozotocin-induced diabetic rats were employed as an animal model. The three-layer electrospun scaffolds containing lignocaine extracts exhibited the highest mean wound closure diameter among the tested groups, surpassing PCL, PCL/collagen, and pure collagen. These findings indicate that the herbal extracts did not affect the physical properties of the scaffolds, but significantly augmented their bioactivity, making them valuable for the treatment of diabetic ulcers (in Figure 5).

### 4.3 Specially structured nanofiber scaffold for diabetic wounds

Specially structured nanofibers exhibit various arrangements, including random orientation (Inai et al., 2005), oriented-ordered structure (Kong et al., 2014), micropatterned structure (Kakunuri et al., 2017) and spider-web-like structure (Kim et al., 2013). These unique fiber structures offer advantages such as excellent adsorption capacity and stable mechanical properties.

Sun et al. (2018) observed a distinctive basketweave-like structure in collagen fibrils of natural skin. Inspired by this, they fabricated biomimetic nanofiber scaffolds with crossed fiber organization via electrospinning. Cell growth experiments comparing the crossed nanofiber scaffolds with aligned and random nanofiber scaffolds were conducted. The results demonstrated that the crossed nanofiber scaffold exhibited superior biocompatibility, enhanced cell growth, and improved cell adhesion compared to the other two groups. Using a diabetic rat wound model, the wound healing effects of the three nanofiber scaffold groups were compared. All three scaffold groups displayed better wound healing than the control group, but the crossed nanofiber scaffold group exhibited the fastest wound closure speed and the most favorable healing state. Additionally, the crossed nanofiber scaffold group exhibited a higher density of newly formed blood vessels.

Sanhueza et al. (2021) developed a dual-size nanofiber scaffold composed of two polymer materials: poly-3-hydroxybutyrate (PHB) and gelatin (Ge). Ge improved the biocompatibility of the scaffold, while PHB crosslinked with Ge, enhancing the mechanical properties and porosity of the scaffold. In cell biological experiments, the double-sized Ge-PHB fiber scaffold effectively promoted fibroblast adhesion, re-epithelialization, and exhibited a slow degradation rate as expected. Furthermore, *in vivo* data demonstrated that the Ge-PHB scaffold resulted in a larger wound healing area, faster wound closure rate, increased formation of new skin tissue, and greater neovascularization content.

Wang J et al. (2022) developed a three-dimensional biomimetic nanofiber scaffold using electrospinning technology and surface modification. The scaffold was designed to collect early biofluids and respond to endogenous electric fields (EF). It exhibited high hydrophilicity and was composed of a polymer matrix of polydopamine and reduced graphene oxide, providing exceptional mechanical properties. The scaffold’s structure functioned as an “electronic skin,” facilitating wound gas exchange, absorbing wound exudates to generate endogenous bioelectricity, and promoting diabetic wound healing.

Jiang et al. (2019) fabricated spacer-oriented nanofiber scaffolds with silicon-doped amorphous calcium phosphate nanocoatings (Si-ACP) on the surface using electrospinning. The substrate materials of the electrospun scaffolds were poly (D, L-lactic acid) (PDLLA) and polycaprolactone (PCL). Scanning electron microscopy (SEM) analysis revealed a random orientation of the nanofibers, with only a small portion exhibiting porosity. The Si-ACP particles were uniformly distributed on the fiber surface. Drug release experiments demonstrated stable release of silicon ions. *In vitro* cellular investigations confirmed the biocompatibility of the scaffolds and their ability to promote cell development. *In vivo* studies utilizing a diabetic mouse wound model showed that compared to a control group using a commercially available dressing, the scaffold group exhibited enhanced wound healing and accelerated neovascularization.

Zhang et al. (2020) utilized coaxial electrospinning to fabricate a specially designed nanofiber scaffold coated with bioglass nanoparticles for improving the healing of diabetic wounds. The therapeutic mechanism of the scaffold involved the controlled release of silicon and calcium ions from the bioglass, which promoted the production of biological factors associated with vascular remodeling in the wound. Additionally, the scaffold’s unique patterned structure provided a larger surface area for fibroblast growth and adhesion, facilitating wound closure. *In vivo* experiments demonstrated that the use of the scaffold increased the number of new blood vessels in the wound and significantly improved the wound closure rate. Furthermore, they prepared an oriented poly (L-lactic acid) (PLLA) fiber scaffold loaded with asiatic acid (AA) through blend electrospinning (Ren et al., 2018). The resulting nanofibers exhibited a porous structure, with AA molecules evenly distributed on the fiber surface. The scaffold exhibited excellent biocompatibility and could regulate cytokine genes involved in macrophage differentiation. *In vivo* experiments using a diabetic mouse wound model showed that compared to the control group, the scaffold group reduced ROS content at the wound site and promoted new skin formation, vascular remodeling, and extracellular matrix (ECM) formation.

Jiang et al. (2020) developed a micro-patterned scaffold by electrospinning and polydopamine coating, loading spherical bioactive glass on the surface. The scaffold matrix material was poly (ε-caprolactone) (PCL). The spherical bioactive glass was uniformly dispersed on the nanofiber surface, enabling stable release of calcium and silicon ions. *In vitro* experiments demonstrated that the scaffold had the potential to promote proliferation and vascular differentiation of human umbilical vein endothelial cells. Further *in vivo* experiments showed that the polydopamine-coated patterned scaffold efficiently promoted the healing of diabetic wounds by enhancing re-epithelialization and collagen deposition. Moreover, the deliberate release of silicon ions from the scaffold had the potential to enhance angiogenesis and suppress inflammation at the wound site, ultimately improving diabetic wound healing.

Su et al. (2020) developed a novel nanofiber scaffold for eradicating bacterial biofilms in diabetic wounds. The scaffold consisted of two components: an electrospun nanofiber scaffold and a soluble microneedle array. The scaffold effectively delivered self-designed antimicrobial peptides both inside and outside the biofilm, resulting in the resistance against a wide range of pathogens. *In vivo* results demonstrated the complete elimination of methicillin-resistant *Staphylococcus aureus* (MRSA) and resistance against *Pseudomonas aeruginosa* by the scaffold.

### 4.4 Electrospinning scaffold as drug-loading platform for diabetic wounds

Currently, conservative treatment options, including drug therapy, are commonly used for patients with diabetes mellitus. Electrospun scaffolds serve as excellent drug-loading platforms, enabling the loading of various conventional drugs for diabetes treatment, such as antibiotics, hypoglycemic drugs, anti-inflammatory drugs, and natural drugs. They can also accommodate bioactive factors, inorganic nanoparticles, specialized biological cells, and other substances that have limited applicability in conventional treatments. Furthermore, in cases where a single drug may not provide optimal wound healing outcomes, electrospun scaffolds can be loaded with multiple drugs, leading to a synergistic therapeutic effect.

#### 4.4.1 Electrospun scaffolds loaded with antibiotics, anti-inflammatory or hypoglycemic drugs

Abbaszadeh et al. (2022) fabricated a cellulose acetate-based nanofiber scaffold loaded with probucol using electrospinning to enhance diabetic wound healing. Different concentrations of probucol-loaded scaffolds were prepared and tested. *In vitro* experiments demonstrated that the scaffold loaded with 1% probucol exhibited superior biocompatibility and cell protective properties. The wound healing ability of the scaffolds was evaluated in a rat model of diabetic excisional wounds. Compared to the drug-free control group, the scaffold group loaded with 1% probucol showed the most significant improvement in wound healing. Gene expression analysis further revealed that the probucol-loaded scaffolds significantly reduced the expression of the glutathione peroxidase gene.

Maashi et al. (2023) developed a collagen-based nanofiber scaffold loaded with Nicaraven using electrospinning technology. The effects of the nanofiber scaffolds loaded with drug concentrations of 2%, 4%, and 6% on diabetic wounds were investigated. *In vitro* assessments demonstrated that the scaffolds were non-toxic to fibroblasts and could mitigate the detrimental effects of oxidative stress on cells. *In vivo* studies using a rat model of diabetic wounds showed a significant increase in wound closure rate, epithelial thickness, and collagen deposition in the scaffold groups with drug concentrations of 4% and 6%.

Zhang et al. (2022) successfully developed a multifunctional nanofiber scaffold loaded with anemoside B4 (ANE) using chitosan (CS) and polyvinyl alcohol (PVA) as polymer matrices. The scaffold exhibited excellent mechanical properties and hydrophilicity, with stable release of ANE. *In vitro* cell experiments confirmed that ANE enhanced the biocompatibility of the scaffold. *In vivo* experiments using a diabetic mouse wound model demonstrated that compared to the blank scaffold group, the ANE-loaded scaffold group reduced the ROS content at the wound site and promoted new skin formation and vascular remodeling.

Lei et al. (2022) reported a cellulose acetate-based nanofibrous scaffold loaded with trilazad mesylate at concentrations of 1%, 3%, and 5%, and investigated the effects of scaffolds with different drug concentrations on diabetic wound healing. The scaffold exhibited good mechanical properties and water absorption, with sustained release of the drug. *In vitro* cell experiments showed that the scaffold with a 3% drug concentration displayed the best biocompatibility. Compared to the control group, the scaffold group exhibited significantly improved wound healing. The scaffold reduced the ROS content at the wound site and promoted the formation of new skin and vascular remodeling.

Tan et al. (2022) fabricated cellulose acetate nanofiber scaffolds incorporating 3%, 5%, and 10% w/w concentrations of pramipexole using electrospinning. The electrospinning technique allowed for the creation of nanoscale diameter fibers and maintained the appropriate pramipexole content. *In vitro* cytotoxicity experiments demonstrated enhanced cell viability for cellulose acetate scaffolds containing 3% w/w pramipexole. *In vivo* experiments using a rat model of diabetic wounds were performed with this scaffold. The results showed that cellulose acetate scaffolds containing 3% pramipexole exhibited faster wound closure rates, increased epithelial thickness, and enhanced collagen deposition compared to scaffolds without the drug and control groups. Gene expression analysis demonstrated that drug-loaded scaffolds significantly reduced oxidative stress and alleviated inflammation.

Kamal et al. (2022) fabricated nanofiber scaffolds using poly (3-hydroxybutyric acid-3-hydroxyvaleric acid) (PHBV) loaded with cefadroxil (CPL) via electrospinning for the treatment of DFU infections. SEM analysis of the scaffold revealed uniform fiber diameter and smoothness. The scaffold exhibited excellent mechanical properties. The drug release profile exhibited an initial burst release followed by a sustained release, lasting up to 2 days. *In vitro* cell experiments demonstrated good biocompatibility and safety of the scaffold, as well as effective antibacterial properties against methicillin-resistant *S. aureus* (MRSA).

Cam et al. (2021) proposed a novel therapeutic strategy involving the preparation of chitosan/gelatin/polycaprolactone nanofiber scaffolds using electrospinning technology. Hypoglycemic drugs, including pioglitazone, metformin, and glibenclamide, were loaded into the polymers to enable the combination of oral hypoglycemic medications. Unlike the conventional solution mixing method, the drugs were loaded into the scaffold under pressure rotation. *In vivo* experiments were conducted using a rat model of diabetic wounds to evaluate the efficacy of the scaffold. The treatment significantly accelerated the healing process of diabetic wounds in type 1 diabetic rats. Additionally, the treatment led to the formation of densely organized collagen fibers in the dermis, improved regeneration of both the dermis and epidermis, and reduced inflammation and edema compared to the group treated with a single drug.

Anand et al. (2022) fabricated nanofibrous scaffolds for diabetic wound healing using polyvinyl alcohol (PVA), sodium alginate (SA), silk fibroin (SF), and loaded with acitretin (AT). The scaffolds demonstrated effective wound healing by maintaining moisture at the wound site. Furthermore, the scaffolds exhibited low cytotoxicity and promoted significant cell migration in human keratinocytes (HaCat) cells, as demonstrated by cell viability analysis and scratch assays, respectively. The scaffolds demonstrated potent antibacterial activity against *P. aeruginosa* and *S. aureus*, as evidenced by successful *in vitro* tests. Moreover, *in vivo* studies conducted on a rat model of diabetes indicated a significant improvement in wound healing when treated with the scaffold compared to the control group that received commercially available wound dressings.

Teaima et al. (2022) reported on the preparation of a nanofibrous scaffold loaded with linezolid using electrospinning. The scaffold was composed of a combination of polyurethane (TPU) and modified chitosan (CS). The morphological and mechanical characteristics of the scaffold were investigated. The results demonstrated that linezolid had no detrimental effect on the TPU/CS morphology. *In vivo* trials conducted on diabetic rats indicated that wound contraction and elastase levels increased in the group treated with the linezolid-loaded scaffold. There was a decrease in plasma nitric oxide and diminished glutathione activity, which facilitated the growth of new skin and remodeling of the vascular system.

Xu et al. (2021) fabricated nanofibrous scaffolds for the treatment of diabetic wounds and enhancement of diabetic wound healing by incorporating Phellodendron Bark extract (Compound Phellodendron Bark Liquid, CPL) into Silk Fibre (SF)/Poly (L-lactic-co-caprolactone) (PLCL) using electrospinning. SEM imaging revealed smooth and beadless nanofibers, with a decrease in fiber diameter observed as the drug concentration increased. The scaffold demonstrated the ability to inhibit methicillin-resistant *S. aureus* (MRSA) and *E. coli* (*Escherichia coli*), enhance proliferation and adhesion of fibroblasts in in vitro cellular experiments. Experiments conducted on diabetic mice demonstrated that CPL-loaded nanofiber scaffolds could increase TGF-β signaling pathway expression and collagen levels, while also inhibiting the expression of pro-inflammatory factors, effectively promoting diabetic wound healing.

#### 4.4.2 Electrospun scaffolds loaded with inorganic nanoparticles, bioactive factors or special active cells

Lv et al. (2017) utilized two-dimensional vermiculite nanosheets as a therapeutic agent to improve diabetic wounds by accelerating neovascularization through the activation of hypoxic-inducible factor 1α (HIF-1α) signaling. They subsequently incorporated the nanosheets into polycaprolactone to produce nanofibrous scaffolds using electrospinning. The experiments demonstrated that the scaffolds can enhance neovascularization, re-epithelialization, and collagen formation in diabetic wounds, thereby facilitating the healing process.

Liu et al. (2022) prepared an electrospun nanofiber scaffold composed of polycaprolactone (PCL)/gelatin/magnesium oxide (MgO) nanoparticles (PCL/gelatin/MgO) to promote wound neovascularization. The main therapeutic mechanism of the scaffold is the controlled release of magnesium ions through magnesium oxide, which influences the expression of biological factors associated with vascular remodeling. Subcutaneous implantation experiments using a rat model demonstrated that this scaffold degrades faster and induces a lower immune response. Within a week of implantation, the PCL/gelatin/MgO scaffolds led to the formation of robust blood vessels. The scaffolds also expedited the healing of diabetic wounds by suppressing inflammatory reactions and promoting angiogenesis and granulation.

**FIGURE 6:**
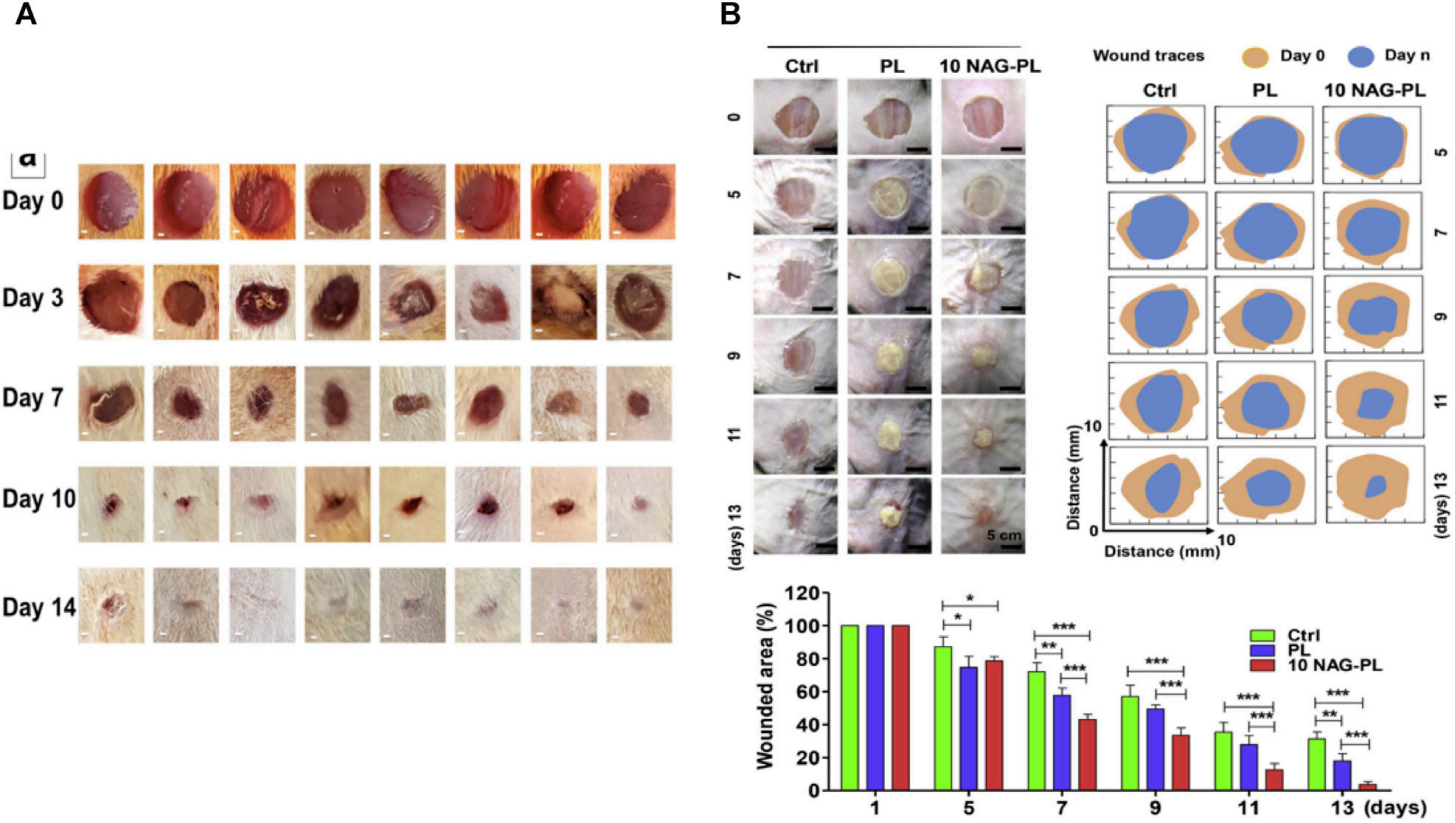
**(A)** Representative wound images from each group on days 0, 3, 7, 10, and 14 after wound creation. On day 3, mild visible wound closure was observed in the treated rats compared to the untreated rats. The treatment groups with drug-loaded scaffolds exhibited significantly faster wound closure on days 7 and 10 compared to the untreated groups and the treatment groups with pure scaffolds (Cam et al., 2021). **(B)** The effect of composite scaffolds containing NAG bioceramic particles on diabetic wound healing. Overview of the size change of excision wounds created in the dorsal skin of diabetic mice over different time periods and traces of wound bed closure for each treatment group *in vivo*. The light brown area indicates the wound area on day 0, and the blue area indicates the wound area on day n (*n* = 5, 7, 9, 11, and 13) (Fan et al., 2022).

Huang et al. (2022b) fabricated a nanofibrillar scaffold of cellulose acetate (CA) through electrospinning. The scaffold was then impregnated with 1.5% w/w puloxamer and subsequently utilized as a drug delivery platform for 30,000 inoculated menstrual blood stem cells (MenSCs). Wound healing assays were conducted using a diabetic rat excisional wound model. The results showed that the wound healing rate was significantly improved in the CA/Puloxamer/MenSCs scaffold group. This group exhibited a significantly higher rate of wound reduction, epithelial thickness, and collagen deposition during the postoperative period. These improvements were attributed to a significantly higher expression level of the vascular endothelial growth factor gene (VEGF) in the scaffold of the treatment group. Fan et al. (2022) prepared PCL/gelatin nanofiber scaffolds loaded with silicate-based bioceramic particles using coaxial electrospinning to promote diabetic wound healing. The NAGEL particles were uniformly dispersed within the PCL/gelatin nanofibers, and silica ions were actively released during scaffold degradation. The nanofiber scaffolds significantly enhanced the adhesion, proliferation, and migration of human umbilical vein endothelial cells (HUVECs) and human keratinocytes (HaCaTs) *in vitro*. *In vivo* experiments were conducted using a rat diabetic wound model. The study revealed that the scaffolds can enhance the processes of angiogenesis, collagen deposition, and re-epithelialization at the wound site by activating the epithelial-mesenchymal transition (EMT) and endothelial-mesenchymal transition (End-MT) pathways *in vivo*, thereby inhibiting inflammatory responses (in Figure 6).

Dwivedi et al. (2018) described a novel polyacrylic resin nanofiber scaffold capable of retaining gentamicin sulfate (GS) and recombinant human epidermal growth factor (rh-EGF). The scaffold was prepared by electrospinning and designed for wound healing in diabetic patients. GS was directly loaded into the polyacrylic resin through electrospinning, while rh-EGF was immobilized on the scaffold through surface modification. *In vitro* cell assays demonstrated that the scaffolds exhibited antimicrobial activity equivalent to pure gentamicin powder. Moreover, *in vivo* experiments conducted on female diabetic mice showed that the scaffolds accelerated the healing process of dorsal wounds compared to the control group that healed naturally.

**FIGURE 7:**
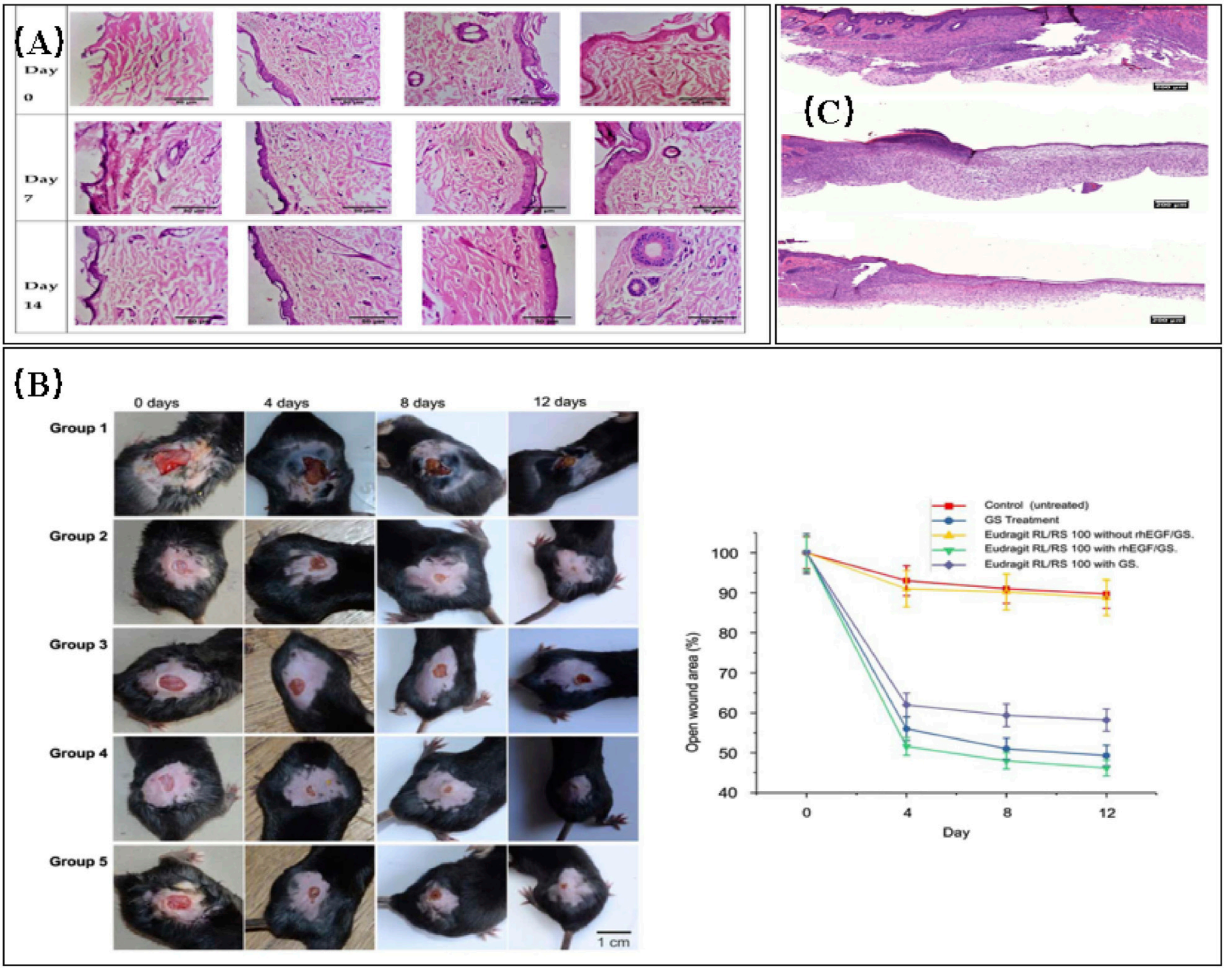
**(A)** Histopathological images comparing normal control, toxic control, F1 (non-crosslinked), and F2 (crosslinked) (Anand et al., 2022). **(B)** Evaluation of wound healing in diabetic C57/BL6 mice treated with different electrospun nanofibrous scaffolds (panels from left to right represent the progression of wound healing over a 12-day period). Group 1: no treatment (negative control); group 2: 0.1% GS ointment (positive control); group 3: Eudragit RL/RS 100 scaffold without GS and rhEGF; group 4: Eudragit RL/RS 100 scaffold with GS and rhEGF; group 5: Eudragit RL/RS 100 scaffold with GS without rhEGF. **(B)** Percentage of open wound area (as a percentage of the initial area) over the 12-day treatment period for each group (Dwivedi et al., 2018). **(C)** Representative histology images of wound sites on day 10. DES: Drug-eluting scaffold; NES: Non-eluting scaffold; Control. The DES group exhibited the most favorable recovery from the cutaneous wound, characterized by a thick epithelial layer (Yin et al., 2016).

Yin et al. (2016) proposed a novel therapeutic approach to convert macrophages from the M1-type to the M2-type. They fabricated drug-eluting nanofiber scaffolds through electrospinning and loaded them with monocyte chemotactic protein-1 (MCP-1) to achieve the desired effect. A stable release of MCP-1 from the scaffold for up to 3 days was observed. *In vitro* studies demonstrated that the electrospun scaffolds had no cytotoxic effects on human keratinocytes. Full-thickness excisional skin wounds were created on diabetic mice for the experiments. The wounds of mice treated with the drug-eluting scaffolds completely healed within 10 days, while those in the control group took 14 days. On day 3, mice treated with the drug-eluting scaffold showed a higher number of M2-type macrophages at the wound site. The significant increase in macrophages is likely the reason for the accelerated wound healing observed (in Figure 7).

Lee et al. (2015) designed a collagen/PLGA nanofiber scaffold incorporating glucophage through hybrid electrospinning for the treatment of diabetic wounds. The release rates of the drug were evaluated *in vitro* and *in vivo* using high-performance liquid chromatography (HPLC). The study utilized a rat model of diabetic wounds. The collagen content in diabetic rats treated with the scaffold was higher than that in control rats treated with commercial dressings, as matrix metalloproteinase 9 (MMP-9) was downregulated. This suggests that the scaffold has the ability to increase collagen content in the treatment of diabetic wounds, effectively promoting early wound healing.

Vijayan et al. (2021) prepared collagen/PLGA/chitosan (CS) nanofiber scaffolds loaded with vascular growth factors. *In vivo* experiments were conducted on a mouse model with streptozotocin-induced diabetes. The study showed that the vascular growth factors, basic fibroblast growth factor (bFGF) and vascular endothelial growth factor (VEGF), bind to heparin through electrostatic interactions. This binding resulted in an acceleration of neovascularization.

Elliott et al. (2019) reported the preparation of two nanofiber scaffolds through electrospinning. One scaffold was composed of collagen and periosteal proteins, while the other scaffold was composed of collagen and Recombinant Human Connective Tissue Growth Factor (CCN2). *In vivo* experiments were performed on a mouse model of diabetic wounds. Both the osteopontin/collagen and CCN2/collagen electrospun scaffolds enhanced the closure rate of excisional wounds compared to collagen alone or untreated wounds. On day 7, both scaffolds showed significantly lower neutrophil infiltration and increased stromal cell infiltration compared to empty and collagen scaffolds. The vascularity of the wound bed increased significantly on day 11. These scaffolds improved the healing rate of full-thickness skin lesions in diabetic mice while simultaneously enhancing angiogenesis.

## 5 Summary and outlook

The global diagnosis rate of diabetes is rapidly increasing, leading to a significant rise in diabetic foot ulcers (DFUs). To meet the needs of patients, continuous innovation in treatment options is necessary to overcome current limitations. Electrospinning scaffolds have shown promising effectiveness in diabetic wound healing and DFU treatment due to their similarity in structure and composition to the extracellular matrix (ECM). These scaffolds provide a conducive environment for cell adhesion, growth, and facilitate skin tissue healing and neovascularization.

This article reviewed the pathological differences in treating normal and diabetic wounds, discussed the pathogenesis of DFUs, and highlighted the urgent need for new treatments. Various electrospun scaffolds with different fluids and structures were introduced, along with their applications in diabetic wounds. Most of these applications promote the regeneration of diabetic wounds through drug-loaded scaffolds or the inherent properties of the electrospun scaffolds, such as stimulating cell migration, promoting angiogenesis, reducing inflammation and infection, and facilitating tissue re-epithelization.

Currently, the therapeutic effects of these electrospun nanofiber scaffolds on diabetic wounds are mainly studied *in vivo* using diabetic rat models. However, animal experiments have limitations when translating to human patients. Overcoming these challenges is crucial to facilitate future clinical trials, including the reduction of toxic residues from organic solvents in nanofibers and minimizing adverse effects associated with drug release. Additionally, industrial production of electrospinning scaffolds is of great importance. Continuous exploration of improving production efficiency, standardizing large-scale production, and fabricating complex nanostructures is needed. Further understanding of the treatment mechanisms of diabetic wound healing and the development of electrospinning technology will contribute to advancements in diabetic wound treatment. Moreover, the introduction of electrospraying, a sister technique to electrospinning, can further expand the possibilities for treating diabetic wounds (Han et al., 2020; He et al., 2022; Zhou et al., 2023a; Chen et al., 2023; Ji et al., 2023; Sun et al., 2023; Xu et al., 2023; Yu and Xu, 2023; Yu and Zhou, 2023). This, combined with research focus and improved patient compliance, holds great promise for improving the current status of diabetic wound treatment.
